# Microextrusion Printing of Hierarchically Structured Thick V_2_O_5_ Film with Independent from Humidity Sensing Response to Benzene

**DOI:** 10.3390/ma15217837

**Published:** 2022-11-07

**Authors:** Philipp Yu. Gorobtsov, Artem S. Mokrushin, Tatiana L. Simonenko, Nikolay P. Simonenko, Elizaveta P. Simonenko, Nikolay T. Kuznetsov

**Affiliations:** Kurnakov Institute of General and Inorganic Chemistry of the Russian Academy of Sciences, 31 Leninsky pr., 119991 Moscow, Russia

**Keywords:** hydrothermal synthesis, V_2_O_5_, VO_2_, acetylacetonate, alkoxoacetylacetonate, work function, gas sensor, microextrusion printing, hierarchical structure, electric conductivity

## Abstract

The process of V_2_O_5_ oxide by the combination of sol-gel technique and hydrothermal treatment using heteroligand [VO(C_5_H_7_O_2_)_2–x_(C_4_H_9_O)_x_] precursor was studied. Using thermal analysis, X-ray powder diffraction (XRD) and infra-red spectroscopy (IR), it was found that the resulting product was VO_2_(B), which after calcining at 300 °C (1 h), oxidized to orthorhombic V_2_O_5_. Scanning electron microscopy (SEM) results for V_2_O_5_ powder showed that it consisted of nanosheets (~50 nm long and ~10 nm thick) assembled in slightly spherical hierarchic structures (diameter ~200 nm). VO_2_ powder dispersion was used as functional ink for microextrusion printing of oxide film. After calcining the film at 300 °C (30 min), it was found that it oxidized to V_2_O_5_, with SEM and atomic force microscopy (AFM) results showing that the film structure retained the hierarchic structure of the powder. Using Kelvin probe force microscopy (KPFM), the work function value for V_2_O_5_ film in ambient conditions was calculated (4.81 eV), indicating a high amount of deficiencies in the sample. V_2_O_5_ film exhibited selective response upon sensing benzene, with response value invariable under changing humidity. Studies of the electrical conductivity of the film revealed increased resistance due to high film porosity, with conductivity activation energy being 0.26 eV.

## 1. Introduction

Nowadays, vanadium (V) oxide is one the most prospective oxide materials for a wide range of applications, owing to its structure and properties. Owing to a combination of layered structure [1] with a large amount of oxygen vacancies and vanadium reactivity in redox reactions, V_2_O_5_ is a very promising material for various applications, such as cathode material for various ion batteries [2,3,4,5,6], a component of antibacterial coatings [7], photocatalysts [8], electrochromic material [9,10,11], charge transfer component in photovoltaics [12,13], etc. In particular, V_2_O_5_ is a prospective receptive layer in gas sensors for the detection of NO_2_ [14,15], NH_3_ [16,17], methane [18], various volatile organic compounds (VOC)—xylene [19], trimethylamine [20], etc. Among these analytes, xylene stands out as a member of the “BTEX” family of compounds—benzene, toluene, ethylbenzene, and xylene. These substances are often used in the petrochemical industry and can often be found in its products, such as fuel, lubricants, grease, etc., as well as in other industries and products. Benzene is one of the more toxic members of the BTEX family, being a dangerous carcinogen and neurotoxin [21], as well as possessing highly flammable vapors. The presence of benzene in exhaled breath can be a tumor biomarker [22]. As a member of the BTEX species, benzene’s presence in the air is an important indicator of the overall degree of its contamination with VOCs [23]. All these factors indicate the necessity of benzene detection.

Almost each of the above-mentioned applications of V_2_O_5_ requires its films to be prepared. There are various ways of oxide films formation, such as chemical deposition from solution (spin-coating [24,25,26,27], dip-coating [28,29]), chemical vapor deposition (CVD) [30,31,32], magnetron sputtering [33,34,35] and just brush deposition [36]. However, these techniques yield gradients in film thickness and functional properties, especially upon scaling from small to large substrate areas. These things considered, all the above-mentioned approaches possess drawbacks upon deposition of films with areas of different composition or properties, which can be necessary for the preparation of sensitive gas sensors of “electronic olfaction” unit type [37] or multicolor electrochromic displays: in order to obtain such films by using the enumerated techniques, masking a part of the surface is required, which constitutes an additional step that makes the deposition process more complicated and potentially influences the properties of already deposited oxides. Printing technologies, such as pen plotter [38,39], ink-jet [40,41,42], microplotter [37], and aerosol [43], allow avoiding this drawback while possessing simplicity and potential for combination with various synthesis approaches, such as sol-gel technology [37,41], hydrothermal synthesis [44,45], etc. In addition to the already mentioned printing techniques, there is a number of others, among which we dedicate special attention to microextrusion printing [46,47,48,49]. Usually, this technique is utilized in creating artificial organs in bioprinting [50], and its application in the formation of inorganic films is insufficiently studied. Microextrusion printing allows obtaining continuous lines (as well as coatings with more complex geometry) with bigger thickness per layer than in the case of ink-jet printing, for example. It is also notable for finer control over ink supply and dosage, inks can be rapidly switched without a cartridge switch, rheological requirements for inks for microextrusion printing are also much less strict, and ink dispensers are cheaper than in the case of ink-jet printing.

Dispersions of various powders can be used as inks for microextrusion printing. Thus, by varying the powder microstructure, one can influence the morphology and properties of the resulting film. Due to their highly developed surface and often due to properties of anisotropy, hierarchic oxide structures show enhanced properties compared to materials that do not exhibit such self-organization [51,52,53,54,55]. At the same time, hydrothermal synthesis is one of the most widespread techniques for hierarchic structure preparation [56,57]. In the case of V_2_O_5_ hydrothermal synthesis, a narrow range of precursors is used: Na_3_VO_4_ [58], (NH_4_)_3_VO_4_ [59], and oxalates [60]. The list of promising precursors for vanadium oxide hydrothermal synthesis can be expanded by adding hydrolytically active heteroligand complexes of [VO(C_5_H_7_O_2_)_2–x_(C_4_H_9_O)_x_] species. Their hydrolysis reaction has been utilized for sol-gel preparation of VO_x_ [61], but, in our opinion, additional hydrothermal treatment after hydrolysis and polycondensation processes initiation for vanadium complexes widely expands our ability to control crystal structure, dispersity, and shape of resulting oxide particles.

Thus, the goal of our research was to study the process of hierarchically structured V_2_O_5_ synthesis by combining sol-gel technology and hydrothermal treatment, as well as to study microstructure, chemosensor, and electrophysical properties of the corresponding oxide film prepared by microextrusion printing.

## 2. Materials and Methods

**Figure 1:**
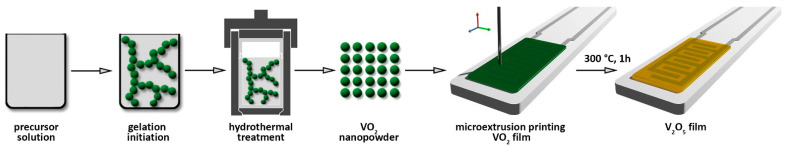
Scheme of V_2_O_5_ powder and film preparation.

The general scheme of oxide powder and film preparation is given in Figure 1.

### 2.1. Oxide Powder Preparation

Hydrolytically active alkoxoacetylacetonate complex [VO(C_5_H_7_O_2_)_2-x_(C_4_H_9_O)_x_] was used as a precursor. The complex was obtained following the procedure described in our [61]. Namely, n-butanol (C_4_H_10_O, 99%, Ekos-1, Moscow, Russia) solution of acetylacetonate [VO(C_5_H_7_O_2_)_2_] (98%, SigmaAldrich, St. Louis, MO, USA) underwent heat treatment for 16 h in an oil bath (bath temperature was 140 °C). The fact that vanadyl alkoxoacetylacetonate had been obtained was verified using ultra-violet (UV) and infra-red (IR) spectroscopy. Afterwards, 1 mL of C_2_H_5_OH (96%, Chimmed, Moscow, Russia) and distilled H_2_O mixture (50% vol. H_2_O) was added to 10 mL of the precursor solution in order to initiate hydrolysis and polycondensation processes. Then, the reaction mixture was transferred into a steel autoclave with Teflon inlay (V = 25 mL), where it further underwent hydrothermal treatment at 200 °C for 2 h. The resulting dark green precipitate was washed with n-butanol several times and dried at 60 °C (3 h). Afterward, oxide powder was annealed in air at 300 °C for 30 min.

### 2.2. Microextrusion Printing of V_2_O_5_ Film

A paste based on oxide powder (dried at 60 °C) and α-terpineol (>97%, Acros Organics, Geel, Belgium) solution of ethylcellulose (48.0–49.5% (*w/w*) ethoxyl basis, Sigma Aldrich, St. Louis, MO, USA) was prepared in order to serve as functional ink. Microextrusion printing of oxide film on the surface of a special Pt/Al_2_O_3_/Pt chip was carried out using a 3D positioning system and pneumatic doser (pressure above ink was 1.1 atm), equipped with dispenser and G27-caliber needle (inner diameter 210 µm). The speed of dispenser movement above the substrate surface was 1 mm/s, the time interval between impulses of paste dosing, as well as their duration, was 0.5 s. The chip consisted of an α-Al_2_O_3_ substrate (R_a_ = 100 nm) with platinum interdigital electrodes on the face side and a platinum meander microheater on the reverse. After printing was finished, the film was dried at 25 °C and then calcined in air at 300 °C for 1 h to remove organic components and facilitate the formation of V_2_O_5_ film.

### 2.3. Powder and Film Characterization

IR-spectroscopy of oxide powder after drying at 60 °C and annealing at 300 °C was recorded in the range of 350–4000 cm^−1^ using InfraLUM FT-08 IR Fourier spectrometer (Lumex, St. Petersburg, Russia), with powder suspensions in nujol mull placed between KBr lenses.

X-ray powder diffraction (XRD) spectra for oxide powder and printed film were obtained using a D8-Advance diffractometer (Bruker, Billerica, MA, USA; CuKα1 = 1.5418 Å, Ni filter, E = 40 keV, I = 40 mA, signal accumulation time 0.3 s/point, resolution 0.02°).

The thermal behavior of dried powder in the temperature range 25–300 °C was studied using simultaneous TGA/DSC analyzer SDT Q600 (TA Instruments, New Castle, DE, USA), with the following regime: heating speed 10°/min, airflow 250 mL/min, with exposure at 300 °C for 30 min.

Oxide powder microstructure, after calcining at 300 °C, as well as V_2_O_5_ film microstructure, was studied using scanning electron microscopy (SEM, two-beam NVision 40 workstation, Carl Zeiss, Inv., Jena, Germany).

Printed V_2_O_5_ film was also studied with atomic force microscopy (AFM) using an NT-MDT Solver PRO microscope (NT-MDT, Zelenograd, Russia), with both surface morphology and local electrophysical properties of the material being characterized. In the latter case, Kelvin Probe Force Microscopy (KPFM) and Scanning Capacitance Microscopy (SCM) were utilized. All techniques were carried out in tapping mode, using ETALON HA_HR probes (ScanSens, Bremen, Germany) with conducting W_2_C film (tip curvature radius ≤ 35 nm). During KPFM, electron work function φ_oxide_ was determined for oxide film. To that end, the sample surface was scanned five times using a probe with already known work function φ_tip_, then for each scan the mean value of contact potential difference φ_CPD_ was determined. Afterwards, φ_oxide_ was calculated as a difference between φ_tip_ and φ_CPD_.

Gas-sensing properties were studied using a specialized precision setup, which has been described in detail in our previous works [47,62]. Sensor responses to H_2_, CO, NH_3_, NO_2_ and benzene (C_6_H_6_) were calculated using the following formula:

S = |R_g_−R_Air_|/R_Air_∙100% (1)

with R_g_ being resistance at given analyte gas concentration and R_Air_—resistance on air. 

The study of electrical conductivity for the V_2_O_5_ film printed on Pt/Al_2_O_3_/Pt chip was carried out by impedance spectroscopy in the temperature range of 50–300 °C using potentiostat-galvanostat with a module for electrochemical impedance measurement (P-45X, Electrochemical Instruments, Russia, Moscow). The frequency interval for measurements was 1 MHz–1 Hz. Chip heating was carried out by applying a voltage to a platinum microheater using a power supply (QJE, PS3003, Ningbo JiuYuan Electronic, Ningbo, China), with control utilizing Testo 868 thermal imager (Testo, Titisee-Neustadt, Germany).

## 3. Results and Discussion

### 3.1. Oxide Powder Characterization

**Figure 2:**
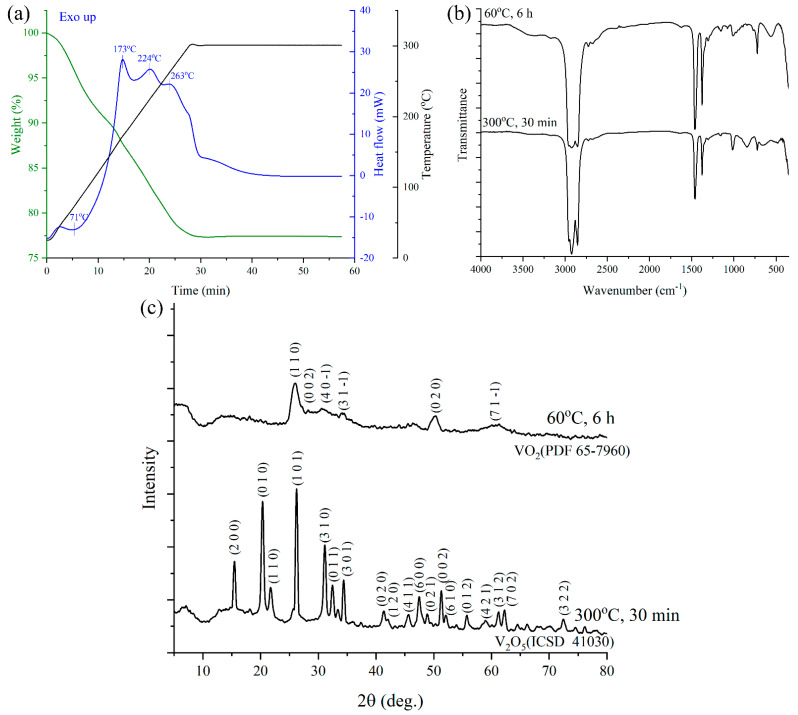
Results of simultaneous thermal analysis for oxide powder after drying: (**a**) IR-spectra for the powder after drying and after annealing at 300 °C; (**b**) Diffractograms for the powder after drying and after annealing at 300 °C (**c**).

First, the thermal behavior of the powder obtained after drying at 60 °C the precipitate formed after hydrothermal treatment was studied. It can be seen from thermograms (Figure 2a) that an endothermic effect starts at 40 °C (2 min), reaching a minimum at 71 °C (5.3 min) and ending around 101 °C (8.3 min) and accompanied by weight loss of about 7%. This can be attributed to the process of gas and organic solvent molecules desorption from the sample surface. Further heating up to 300 °C (~28 min) is accompanied by a linear decrease in weight. Several exothermic effects are observed on the differential scanning calorimetry (DSC) curve at the same time, with maxima at 173, 224, and 263 °C. These peaks can be attributed to a combination of oxidation of organic species in the sample and transformation from VO_2_(B) to VO_2_(R). After reaching 300 °C, a broadened exothermic effect occurs around 31 min, accompanied by a small (~0.15%) gain in weight. The weight gain points to the process of VO_2_ oxidation to V_2_O_5_ occurring at that moment since V_2_O_5_ has more oxygen per metal ion than VO_2_ and oxygen infiltration into the sample thus must accompany oxidation. As can be seen from the thermogravimetry analysis (TGA) curve, further holding the sample at 300 °C results in powder weight stabilization, with net weight loss being 22.6% for the full temperature range.

IR-spectroscopy results for oxide powder after drying at 60 °C and after annealing at 300 °C show (Figure 2b) that hydrothermal treatment of reactive mixture yielded vanadium dioxide, which oxidized to pentoxide after further annealing at 300 °C. This is well demonstrated by changes in IR spectra in the range of 450–1050 cm^−1^. Two bands related to V = O bonds vibrations can be seen for powder after drying, with maxima at 1007 and 561 cm^−1^ [63]. After annealing at 300 °C shapes of the band around 1000 cm^−1^ changed, a band with a maximum of 658 cm^−1^ replaced the one with a maximum at 561 cm^−1^, and a new band, characteristic of bridge V-O-V bonds in V_2_O_5_, appeared at 842 cm^−1^ [63].

X-ray powder diffraction results (Figure 2c) confirm the data from IR-spectroscopy. Reflexes in hydrothermal treatment products can all be attributed to the monoclinic VO_2_(B) phase (PDF card 65–7960), with small intensity and large width of the peaks hinting at the high dispersity of prepared vanadium dioxide. Full profile analysis showed that the mean coherent scattering regions (CSR) size were 3.3 ± 1.4 nm, which indicates high dispersity for the product but can also be a result of its low crystallinity. Diffractogram for the powder annealed at 300 °C (30 min) is in good agreement with Inorganic Crystal Structure Database ICSD #41030 pattern and corresponds to orthorhombic V_2_O_5_ (space group Pmn21). The mean CSR size, in this case, was 20.3 ± 5.5 nm.

**Figure 3:**
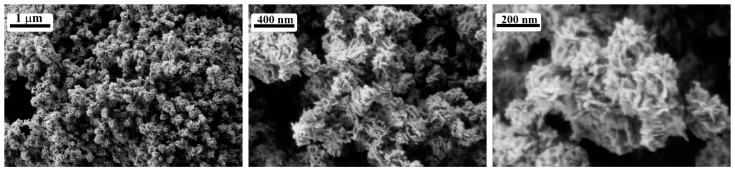
SEM results for oxide powder after annealing at 300 °C.

SEM data (Figure 3) shows that after annealing at 300 °C, particles of as-prepared pentoxide have the shape of nanosheets with a thickness of about 10 nm and length of 50–60 nm, assembled in hierarchical structures about 200 nm in size with well-developed surface. Particle sizes thus determined is in agreement with previously calculated mean CSR for V_2_O_5_ (~20 nm).

### 3.2. V_2_O_5_ Film Characterization

**Figure 4:**
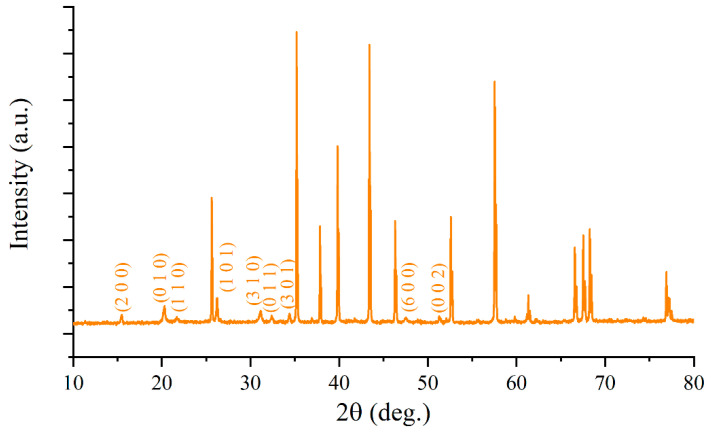
Diffractogram for V_2_O_5_ film on Pt/Al_2_O_3_/Pt chip.

After microextrusion printing of the oxide film and annealing it at 300 °C, its phase composition was first studied. It can be seen from the recorded diffractogram (Figure 4) that the crystal structure of orthorhombic V_2_O_5_ (space group Pmn21; ICSD #41030) formed. Other intense peaks correspond to Pt/Al_2_O_3_/Pt chip materials. Thus, the steps of paste preparation, oxide film printing, and the following annealing did not result in the appearance of any admixtures.

**Figure 5:**
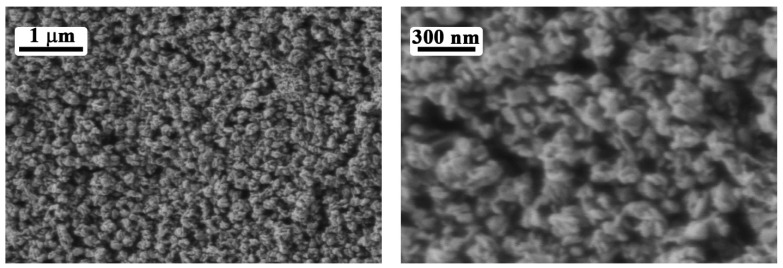
Microstructure for the printed V_2_O_5_ film (according to SEM).

SEM results (Figure 5) indicate that the resulting oxide film, as well as the source powder, consists of hierarchically assembled structures based on nanosheets. The film is uniform, does not have any deficiencies such as cracks or delamination, and possesses heightened porosity. The mean size of these agglomerates is 200 ± 15 nm, which correlates well with the utilized oxide powder. No admixtures with microstructure differing from the one exhibited by vanadium (V) oxide particles were found in the film. 

**Figure 6:**
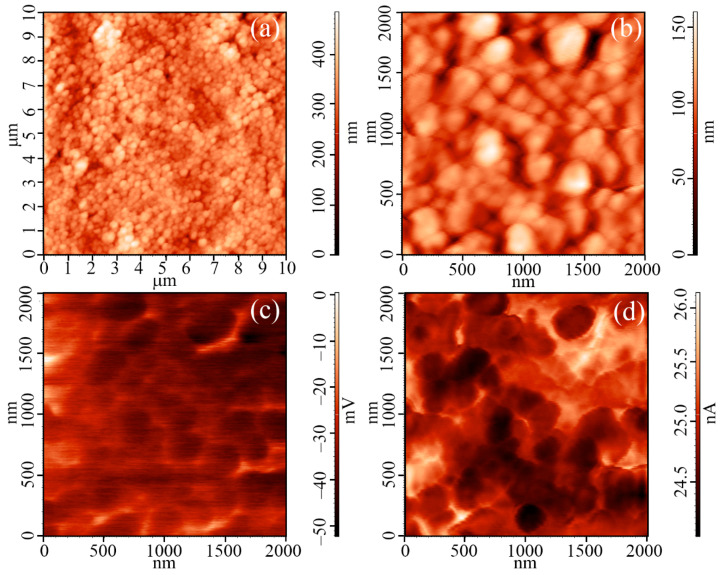
AFM results for the printed V_2_O_5_ film: Topography (**a**,**b**), maps of potential surface distribution (**c**), and gradient in capacity of “probe tip-sample surface” capacitor distribution (**d**).

AFM results (Figure 6) confirm data obtained with SEM. Round-shaped particles with a diameter of about 220 ± 15 nm are seen on topographic scans (Figure 6a,b). Their size is close to the diameter of the hierarchic oxide structures estimated using SEM (200 ± 15 nm). It can be seen from the topographic scan with an area 100 µm^2^ (Figure 6a) that the film is quite uniform: even though the maximum height difference is 483 nm, the mean squire roughness is just 43 nm. In addition to surface topography, the local electrophysical properties of the film were studied during AFM experiments using KPFM and SCM techniques. KPFM results (Figure 6c) show that surface potential is uniformly distributed across the studied surface (the difference between minimal and maximal values of contact potential is just 50 mV), although a small increase in its value is observed on grain boundaries, which is also evident from SCM results (Figure 6d). This implies a shift of charge carrier density and charged deficiencies into the boundary between separate hierarchic structures. Using KPFM, the value of φ_oxide_ was determined for V_2_O_5_ film, which was 4.81 eV. It is well known that V_2_O_5_ film which has been exposed to air can have work function values in the range of 4.7–5.3 eV due to V^4+^ presence. Thus, our value of φ_oxide_ is in good agreement with the literature data. At the same time, a comparison of the work function for our film with those available in the literature reveals that in our case value (4.81 eV) is lower than in cases when vanadium pentoxide was prepared by vanadium (V) oxyisopropoxide VO(C_3_H_7_O)_3_ (5.3 eV [64], 5.1 eV [13]), the reaction between metallic vanadium with H_2_O_2_ (5.4 eV [65]), thermal treatment of Na_2_VO_4_ solution (5.2 eV [66]), vanadium acid condensation (5.15–5.5 eV [67]), physical deposition from gas phase upon evaporating V_2_O_5_ (5.1 eV [12]), but a little higher, than for vanadium pentoxide prepared by hydrothermal synthesis using sodium vanadate [58]. It is generally considered that the work function for materials based on V_2_O_5_ is, to a large extent, determined by V^4+^ cations and oxygen vacancies content [12,68]. For example, it was shown in work [68] that a decrease in the number of oxygen vacancies and V^4+^ ions result in increased work function (from 4.73 to 5.01 eV in the referenced study). This allows assuming that in our material number of oxygen vacancies and V^4+^ ions are larger than in the case of synthesis from vanadium(V) compounds, except for hydrothermal synthesis, where it would seem a partial reduction in V^5+^ to V^4+^ occurs in the reaction mixture. It should be noted that the work function for our material differs by ~0.1 eV from the one for vanadium oxide we earlier prepared in [61] (4.89 eV), where we utilized sol-gel technique and the same [VO(C_5_H_7_O_2_)_2–x_(C_4_H_9_O)_x_] precursor. Considering that the annealing regime for obtained vanadium oxide films in both studies is the same, one can assume that additional hydrothermal treatment of the reaction mixture after hydrolysis initiation yields a higher amount of deficiencies than in the case of sol-gel.

### 3.3. Sensory Properties of V_2_O_5_ Film

The prepared V_2_O_5_ film exhibits relatively high conductivity in a wide temperature range. Out of all used analytes, the highest response was obtained for benzene (at 300 °C).

**Figure 7:**
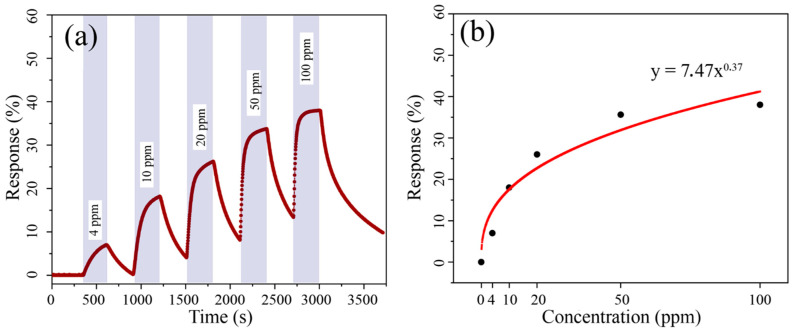
Experimental curves of changes in sensing response during detection of benzene in the concentration range of 4–100 ppm (**a**); Dependence of sensing response to benzene from its concentration (**b**).

**Figure 8:**
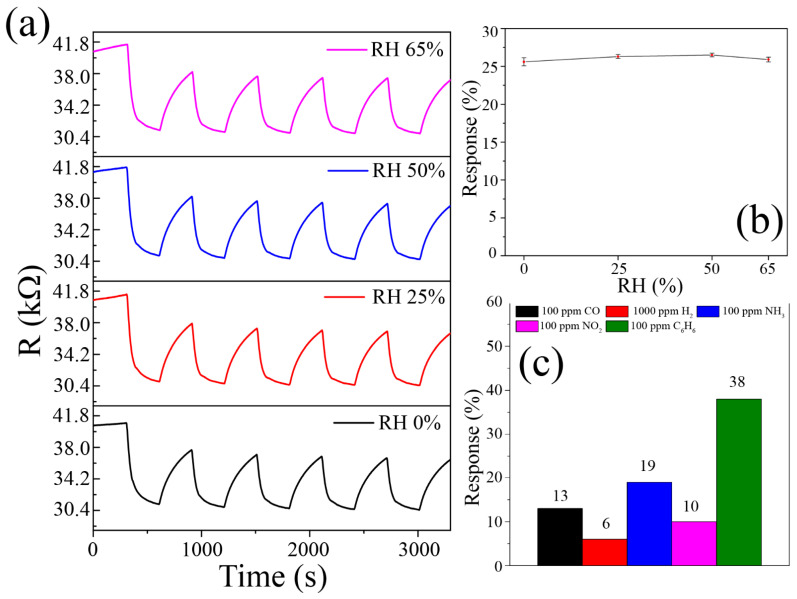
Experimental curves of resistance change upon sensing 20 ppm of benzene at varying relative humidity (**a**); Dependence of sensing response to 20 ppm of benzene from relative humidity (**b**); Selectivity diagram: sensing responses to various analyte gases at 300 °C (**c**).

Upon studying the properties of oxide film for sensing benzene (4–100 ppm) at 300 °C (Figure 7a), it was determined that increase in concentration results in a consecutive decrease in film resistance compared to baseline and an increase in response (S) from 7 to 38%. The dependence of response (S) on benzene concentration (Figure 7b) is well described by the Freundlich equation: *S = kCa*, with *k* and *a* being proportional and exponential constants, which correspond to adsorption capacity and adsorption intensification, respectively [69]. In our case the equation is as follows: *S = 7.47x^0.37^*, with a determination coefficient (R^2^) of 89%. This dependence is typical of chemoresistive gas sensors and is in good agreement with the literature’s data [70,71]. The reproducibility of sensing responses upon detecting 20 ppm of benzene in the atmosphere with varying (0–65%) relative humidity (RH) was also studied (Figure 8a). It was established that resistance values are identical at varying humidity, which is unusual for semiconductor receptive materials in chemoresistive gas sensors. Humidity does not affect either the value of operating resistance or the response value (Figure 8b). Thus, printed V_2_O_5_ film exhibits the same behavior at different humidity, which is important for practical application.

The mechanism for detecting benzene can be described using the generally accepted views on reactions that occur between gas and semiconductor surfaces [72,73,74,75]. In ambient air at elevated temperatures, adsorption of oxygen molecules on the semiconductor surface occurs, which results in changes in material resistance due to electrons from the conduction band reducing O_2_ to ionic species (reaction 2). Depending on operating temperature, various ion-sorbed species can form O^2–^, O^–^, and O_2_^–^ [76]. At moderate operating temperatures (in particular, 300 °C), O^–^ particles are most likely formed. The presence of such ions on the semiconductor surface facilitates the formation of core-shell type electron structure. Inner regions of semiconductor particles become the core, while the electron depletion layer (EDL) on the surface [77], formed due to the consumption of electrons for O_2_ to O^−^ reduction becomes the shell. Interaction with benzene occurs with a redox reaction on the semiconductor surface between O^−^ and organic gas, with the latter undergoing oxidation (reaction 3).

O_2_ + 2e^−^ ↔ 2O^−^_ad_ (2)

C_6_H_6_ + 7/5O^−^_ad_ ↔ 6CO_2_ + 3H_2_O + e^−^ (3)

Thus, the freed electrons enter the V_2_O_5_ conduction band, resulting in resistance change (Figure 8a), which allows registering resistive response. There is a number of studies in the literature [72,73,75] where vanadium oxide was used as a component of sensors for detecting volatile organic compounds (VOCs) at moderate and high operating temperatures, which can be attributed to its catalytic activity in the oxidation of these gases due to high concentration of adsorbed oxygen species on its surface [19,78].

Another factor influencing the sensing properties is the presence and number of oxygen vacancies since they can facilitate the formation of oxygen species on the film surface, which would then react with organic gas and release additional electrons to the material conduction band, resulting in increased conductivity change and response [79]. These processes can be described by the following equations [79]:

O_V_ + O_2_ (gas) ↔ O_2_^−^_ad_ + O_V_^*^ (4)

2O_V_ + O_2_ (gas) ↔ O_2_^2−^_ad_ + 2O_V_^*^↔ 2O^−^_ad_ + 2O_V_^*^ (5)

Where O_V_^*^ is a single electropositive oxygen vacancy. As described earlier, the work function value for our material indicates a relatively large number of vacancies, which most likely positively affects sensing response and increases selectivity to benzene. 

In humid air, competition between water molecules and other gases occurs for adsorption onto the oxide surface and reaction with oxygen species [21,80,81], with water forming hydroxyl groups in the course of the following reaction [81]:

H_2_O + O^−^_ad_ ↔ 2 OH + e^−^ (6)

Thus, it seems unlikely that oxygen vacancies significantly influence this process by themselves. There are reports, however, that show that the presence of redox-active species, such as Pr^3+^/Pr^4+^ or Ce^3+^/Ce^4+^, results in practically constant sensing response under varying humidity [81,82]. In our case, a high number of V^4+^ ions (accompanying oxygen vacancies) might have the same effect by reducing hydroxyl groups back into water and turning into V^5+^, which then is reduced back into V^5+^ by electrons from V_2_O_5_:

V^4+^ + 2OH ↔ V^5+^ + H_2_O + O^−^_ad_ (7)

V^5+^ + e^−^ ↔ V^4+^ (8)

The plausibility of such a mechanism is confirmed by a number of studies, in which it was shown that hydroxyl formation is intermediate, and the final product is vanadyl bond V = O with V^5+^ [83,84]. It should be noted that in [75], where V_2_O_5_ is used to detect xylene (another member of BTEX with a similar sensing mechanism), response changes noticeably in the range of 30–90% RH, dropping by 21% at 90% RH (and ~16% at 65% RH). That might imply that, in our case, there is a larger number of oxygen vacancies and accompanying V^4+^ ions in V_2_O_5_. 

As a result of chemosensing measurements, a selectivity diagram for detecting 100 ppm CO, NH_3_, NO_2_, C_6_H_6,_ and 1000 ppm H_2_ at 300 °C was built (Figure 8c). Selectivity to benzene is evident, with response to other gases not exceeding 19%.

Data in Table 1 allows comparing the sensing properties of our material with those reported in the literature. The comparison highlights the main advantage of our material, namely its constant response values across the studied RH range. This shows that our material is promising for practice all application.

### 3.4. Electric Conductivity of V_2_O_5_ Film

**Figure 9:**
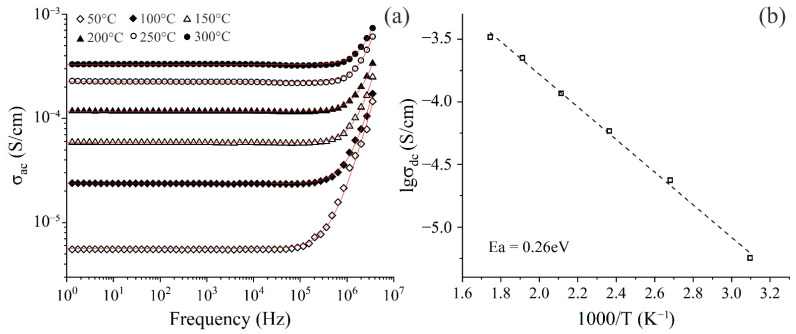
Frequency dependencies of the printed V_2_O_5_ film conductivity at various temperatures (**a**); Temperature dependence of conductivity for the film (**b**).

Analysis of frequency dependencies of electric conductivity (Figure 9a) for the studied film reveals two regions—the frequency-independent range (plateau from 1 to ~10^5^ Hz) and frequency-dependent range, where an increase in conductivity is observed with an increase in frequency (from 0.1 MHz and higher). The former is caused by the diffusion of mobile ions in the sample originating from applying alternating current and corresponds to the direct current conductivity σ_dc_, which depends only on temperature. At a certain point, conductivity begins to increase with an increase in frequency, revealing a dispersion region that is attributed to the hopping mechanism of charge carrier transport [90]. It can be seen that an increase in temperature leads to a shifting of dispersion region to higher frequencies and an increase in alternating current conductivity σ_ac_, which indicates the semiconducting nature of the material [91]. It is known that the conductivity of such materials possesses thermally activated character, i.e., higher temperatures lead to an increase in charge carrier mobility and lower semiconductor resistance [92]. The observed dependence of electrical conductivity on frequency and temperature is well described by Jonscher power law (9):

σ_ac_(ω,T) = σ_dc_(T) + Aω^s^ (9)

With *σ_ac_* being the conductivity value found from the real part of the total conductivity measured for alternating current, *σ_dc_*—conductivity for direct current, *ω*—angular frequency of the applied electric current, *A* and *s*—fitting coefficients determined by the nature of the studied material and dependent on temperature and frequency. In general, *s* has values between 0 and 1, although in some cases, *s* can be larger than 1.

Determining electrical conductivity values for direct current, calculated using Jonscher law, were further used for evaluating temperature dependence of conductivity (Figure 9b), as well as for calculating its activation energy by Arrhenius Equation (10):

σ_dc_ = σ_0_·exp(-E_a_/k_B_T) (10)

with *σ_0_, k_B_, E_a_* being a preexponential factor, Boltzmann constant, and activation energy for conductivity, respectively.

In the studied temperature range activation, the energy value for V_2_O_5_ film was 0.26 eV, which is comparable to *E_a_* values for materials of similar composition [92]. However, conductivity values are lower than those encountered in the literature for planar materials based on vanadium pentoxide [93,94]. This can be attributed to the high porosity of the printed film, which results in the decreased contact area between grains, raising material resistance.

## 4. Conclusions

As a result of our research, the process of synthesizing hierarchically assembled V_2_O_5_ by a combination of sol-gel technique and hydrothermal treatment using heteroligand [VO(C_5_H_7_O_2_)_2–x_(C_4_H_9_O)_x_] precursor was studied. It was shown that the prepared oxide nanopowder of V_2_O_5_ with orthorhombic crystal structure consists of hierarchic structures with a diameter of ~200 nm, assembled from nanosheets with a length of 50–60 nm and thickness of ~10 nm. By using functional inks based on oxide nanopowder, V_2_O_5_ film with a similar microstructure was formed by microextrusion printing. The work function value (4.81 eV), evaluated using KPFM, allows us to assume that the chosen synthesis technique and precursor species yield an increased number of oxygen vacancies and V^4+^ ions in the material. The chemoresistive gas sensor based on the printed V_2_O_5_ film exhibits selectivity to benzene at 300 °C. Sensing response, upon an increase in benzene concentration in the range of 4–100 ppm, rises from 7 to 38%. It should be noted that, at the same time signal does not depend on humidity. A study of the temperature dependence of printed V_2_O_5_ film showed that activation energy value (0.26 eV) is comparable to values for similar composition materials, while electric conductivity is a little lower due to the increased porosity of the printed film.

Thus we have shown that hydrolytically active heteroligand complexes of [VO(C_5_H_7_O_2_)_2–x_(C_4_H_9_O)_x_] composition is a promising precursor for the synthesis of hierarchic V_2_O_5_ nanostructures by the combination of sol-gel technique and hydrothermal treatment, and microextrusion printing allows to efficiently form thick films based on vanadium (V) oxide with the developed surface as a receptive layer for resistive gas sensors to benzene, stable under humidity fluctuations.

## Figures and Tables

**Table 1:** Comparison of benzene gas sensing properties of prepared V_2_O_5_ film and several previously reported sensors.

Material	Operating Temperature, °C	C_6_H_6_ Concentration, ppm	Response S	Humidity Influence	Reference
Cr/ITO	210	166	~27%	N/A	[85]
4%Pd/TiO_2_/ MoS_2_ composite	Room temperature	50	65%	N/A	[86]
CoPP-TiO_2_^1^	327	9	~83%	RH↑ => S↓ ^2^	[87]
ZnO/Au rose-like structures	206	20	~94%	N/A	[88]
0.5%Pt/ZnO spheres	250	100	~94%	N/A	[69]
ZnO/BN	300	100	~23%	S changes nonmonotonically	[21]
Co_3_O_4_ hierarchical structures	160	50	~105%	N/A	[89]
Bi-SnO/rGO	150	5	~98%	RH↑ => S↓	[80]
V_2_O_5_ hierarchic structures	300	100	38%	Constant S	This work

^1^ Cobalt porphyrin-functionalized TiO_2_; ^2^ Humidity influence studies performed on toluene.

## Data Availability

Not applicable.
